# The Long-Term Impact of a Teleprehabilitation Programme on Modifiable Risk Factors and Quality of Life After Cardiac Surgery

**DOI:** 10.1093/ejcts/ezag207

**Published:** 2026-08-06

**Authors:** Lieke van Susante, Loes Janssen, Elham Bidar, Gerrit Slooter, Peyman Sardari Nia

**Affiliations:** Department of Cardiothoracic Surgery, Heart and Vascular Center, Maastricht University Medical Center, Maastricht 6202 AZ, The Netherlands; Department of Surgery, Máxima Medical Center, Veldhoven 5504 DB, The Netherlands; Department of Cardiothoracic Surgery, Cardiovascular Research Institute Maastricht (CARIM), Maastricht University, Maastricht, The Netherlands; Department of Surgery, Máxima Medical Center, Veldhoven 5504 DB, The Netherlands; Department of Cardiothoracic Surgery, Heart and Vascular Center, Maastricht University Medical Center, Maastricht 6202 AZ, The Netherlands; Department of Cardiothoracic Surgery, Cardiovascular Research Institute Maastricht (CARIM), Maastricht University, Maastricht, The Netherlands; Department of Surgery, Máxima Medical Center, Veldhoven 5504 DB, The Netherlands; Department of Cardiothoracic Surgery, Heart and Vascular Center, Maastricht University Medical Center, Maastricht 6202 AZ, The Netherlands; Department of Cardiothoracic Surgery, Cardiovascular Research Institute Maastricht (CARIM), Maastricht University, Maastricht, The Netherlands

**Keywords:** teleprehabilitation, cardiac surgery, modifiable risk factors, quality of life

## Abstract

**Objectives:**

To assess the long-term effects of teleprehabilitation on modifiable risk factors and quality of life up to 1 year following elective cardiac surgery.

**Methods:**

This secondary analysis of the Digital Cardiac Counselling trial, a randomized controlled trial, compared multimodal teleprehabilitation with standard care in patients undergoing elective cardiac surgery. The teleprehabilitation programme targeted physical fitness, inspiratory muscle training, psychological support, nutritional optimization, and smoking cessation. Outcomes included trajectories of modifiable risk factors and quality of life measured preoperatively and at 3, 6, and 12 months postoperatively. Generalized linear mixed models assessed differences over time between groups, with exploratory post hoc analyses for individual timepoints.

**Results:**

Both groups showed postoperative reductions in all assessed modifiable risk factors and improvements in quality of life over time (*P* < .001), except for nutritional optimization (*P* = .173). Teleprehabilitation was associated with more favourable trajectories for smoking behaviour (*P* = .045) and nutritional risk (*P* = .011), with consistently lower incidences throughout follow-up. Smoking prevalence remained significantly lower in the teleprehabilitation group at all timepoints (*P* < .05), while malnutrition prevalence was lower at 3 months postoperatively (*P* = .012). Quality-of-life scores were consistently higher in the teleprehabilitation group throughout follow-up (*P* = .002).

**Conclusions:**

Teleprehabilitation in cardiac surgery patients not only reduces postoperative complications, but may also provide additional long-term benefits, particularly in smoking cessation, nutritional optimization, and quality of life. These findings support teleprehabilitation as a valuable addition to standard preoperative care, with possible sustained benefits for long-term lifestyle and quality of life.

## Graphical Abstract

**Figure:**
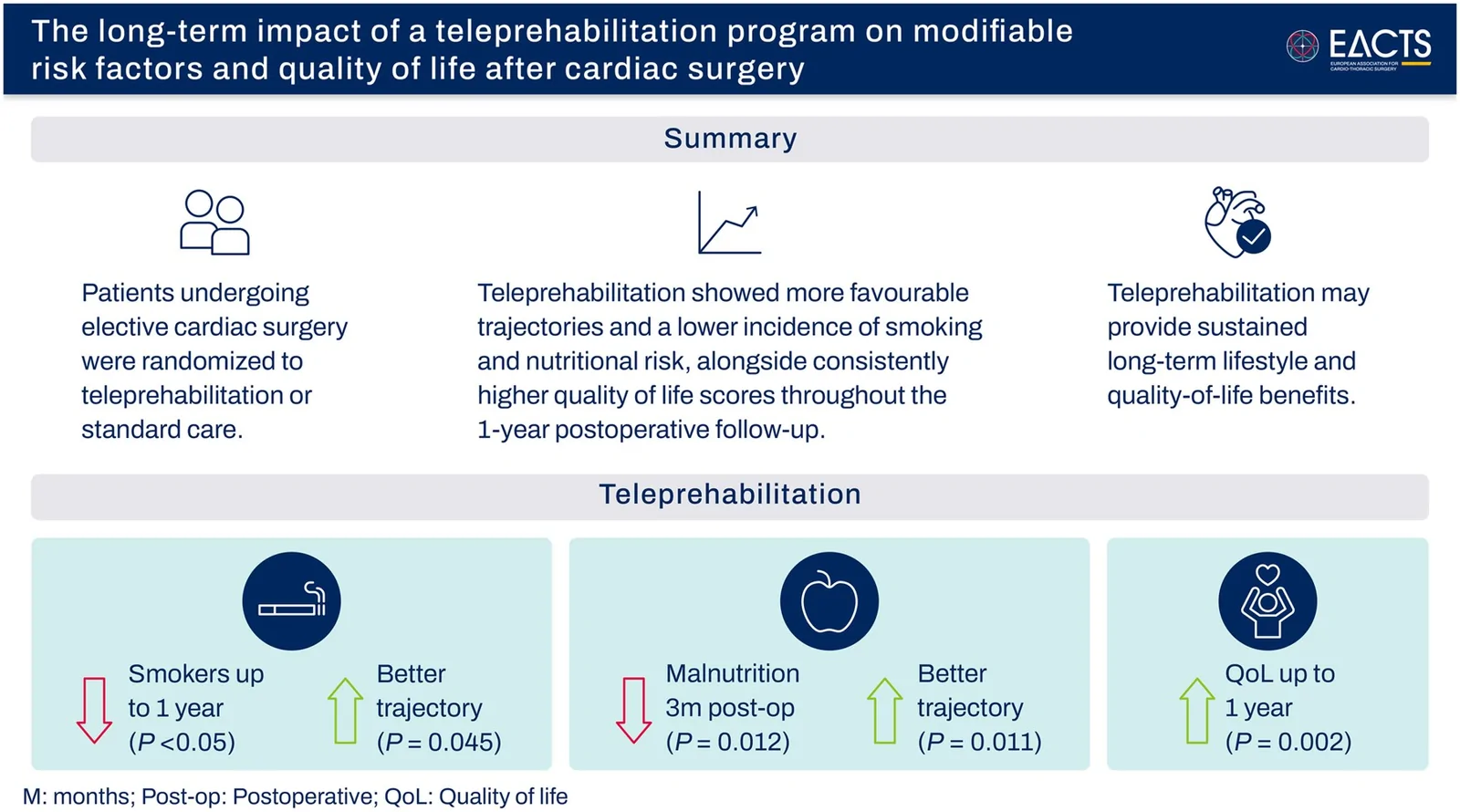

## Introduction

Patients undergoing cardiac surgery present with a high burden of modifiable risk factors,^1^ which are associated with postoperative morbidity, prolonged hospital stays, and high readmission rates.^2–5^

Prehabilitation has emerged as a promising strategy to improve postoperative outcomes by using the preoperative waiting period to address modifiable risk factors through targeted multimodal interventions, including functional exercise training, smoking cessation, and psychological support.^5–11^ The recently published Digital Cardiac Counselling (DCC) trial, a multicentre randomized controlled trial, demonstrated that a personalized multimodal teleprehabilitation programme reduced the composite end-point of major adverse cardiac events (MACEs) within 1 year in patients undergoing cardiac surgery.^12^ The teleprehabilitation group also showed a reduction in modifiable risk factor incidence in the preoperative period and higher quality of life scores 1 year after surgery. However, the durability of these improvements beyond the preoperative period and the trajectories of quality of life throughout the first postoperative year have not been established.

Teleprehabilitation leverages the prospect of cardiac surgery as a window of heightened patient motivation for behavioural change.^1^^,^^13^ While individual preoperative interventions have demonstrated short-term benefits, including improved functional capacity,^6^^,^^8^^,^^9^ reduced depressive symptoms,^14^ and increased smoking cessation,^15^^,^^16^ evidence for sustained long-term effects in cardiac surgery patients is limited. Moreover, these interventions have been evaluated in isolation and face-to-face, in contrast to teleprehabilitation, which integrates multiple components into a comprehensive multimodal programme delivered digitally at home.

Long-term reduction of risk factors is clinically relevant, as their persistence or recurrence after surgery increases the risk of future cardiovascular disease. To our knowledge, no previous study has examined the long-term effect of teleprehabilitation on modifiable risk factors in cardiac surgery patients.

In this secondary analysis of the DCC trial, we assessed the effect of teleprehabilitation on modifiable risk factor incidence and the trajectory of quality-of-life scores up to 1 year after elective cardiac surgery.

## Methods

### Study design

The DCC trial was an open-label, multicentre, randomized controlled trial. The study protocol and results have been published previously.^12^^,^^17^^,^^18^ In summary, adult patients from Maastricht University Medical Centre+ and multiple referral centres undergoing elective cardiac surgery were randomized in a 1:1 ratio to teleprehabilitation or standard care. The teleprehabilitation group received a personalized multimodal teleprehabilitation programme based on individual risk profiles. All patients provided written and telephone-based informed consent. The trial was registered at clinicaltrials.gov (NCT04393636) and approved by the Medical Ethics Committee Maastricht in April 2020 (NL73754.068.20/METC20-028).

### Patients

Adult patients scheduled for elective cardiac surgery were screened for eligibility. Surgery needed to be scheduled at least 8 weeks after consent, allowing a minimum prehabilitation time of 6 weeks. Detailed eligibility criteria and reasons for non-participation have been published elsewhere.^17^^,^^18^

### Multimodal teleprehabilitation programme

At baseline, all patients were screened for modifiable perioperative risk factors. Patients in the teleprehabilitation group who tested positive for one or more of the risk factors could participate in the corresponding modules, selected through shared decision-making with a case manager.

The teleprehabilitation programme comprised 5 modules: functional exercise training, inspiratory muscle training, nutritional support, psychological support, and smoking cessation. It was delivered by a multidisciplinary team, including a cardiologist, nurses, physiotherapists, a counselling psychologist, and dietitians exclusively via an online platform supporting video consultations and module-specific information.

All patients, regardless of allocation, received access to the platform providing audiovisual information about their care pathway and upcoming surgery. The platform was also used to collect questionnaire-based data on risk factor incidence and quality of life through 12 months postoperatively.

Assessment occurred at baseline, at hospital admission, and at 3, 6, and 12 months after surgery.

Detailed module descriptions are available in the published trial design.^17^

### Modifiable risk factors

#### Functional exercise training

Patients screening positive for reduced physical fitness using a subset of the 36-item Short Form Health Survey (SF-36), or scoring ≥6 on Visual Analogue Scale (VAS) questions regarding physical limitations, knowledge of physical limits, or anxiety about physical exercise, were offered the functional exercise training module, consisting of video call physiotherapy counselling and a home-based exercise programme.

#### Inspiratory muscle training

Patients with a pulmonary risk score ≥2, an index assessing postoperative pulmonary complications risk based on age, body mass index (BMI), smoking status, and a chronic obstructive pulmonary disease (COPD) history,^19^ were offered inspiratory muscle training using the POWERbreathe device, performed 5 times per week, twice daily, progressing from 30% to 55% of predicted maximal inspiratory pressure over 6-8 weeks.

#### Psychological support

Patients scoring ≥8 on either subscale of the Hospital Anxiety and Depression Scale (HADS) were offered least 3 sessions with a psychological counsellor based on acceptance commitment therapy and mindfulness principles.^20^

#### Nutritional support

Patients with obesity (BMI ≥30 kg/m^2^) or malnutrition risk (Malnutrition Universal Screening Tool [MUST] score ≥2^21^) were offered dietary counselling and, where indicated, protein-enriched supplementation.

#### Smoking cessation

Patients who smoked at baseline were offered a structured smoking cessation programme led by a pulmonary care nurse, incorporating motivational interviewing and, where appropriate, pharmacological support. Smoking status was self-reported at each timepoint. Cessation was defined as self-reported abstinence.

### Study outcomes

The primary outcome was the incidence of modifiable risk factors at 3, 6, and 12 months after surgery, defined as the percentage of patients meeting the cutoff value for each module. All patients screening positive for a given risk factor were included in that factor’s analysis, regardless of module participation. Preoperative incidences have been reported previously.^12^ The secondary outcome measure was quality of life, assessed using the EQ-5D-5L (5-Level EQ-5D) at each timepoint. Responses across the 5 dimensions (mobility, self-care, usual activities, pain/discomfort, and anxiety/depression) were converted to one utility index score.

All baseline characteristics of the participants were obtained from the hospital’s electronic health records, except for the New York Heart Association (NYHA) class and Canadian Cardiovascular Society (CCS) class, which were patient reported and obtained from the online platform. Socioeconomic status (SES) scores per neighbourhood were obtained via Statistics Netherlands, which were measured based on household data regarding welfare, educational level, and labour participation. Scores range from −1 (lowest SES) to +1 (highest SES), where 0 is the average SES in the Netherlands.^22^

### Missing data

Patients who did not complete any assessment were excluded from the analysis; those completing at least one assessment were included. Due to a technical error in the data collection platform, VAS questions were not distributed to the intervention group at any postoperative timepoint. These outcomes were therefore excluded from analyses.

### Statistical analysis

Baseline characteristics are presented for the total study population and per group. Continuous variables are expressed as mean ± standard deviations or medians with interquartile ranges (IQRs), and categorical variables as absolute numbers and percentages.

The effect of teleprehabilitation on modifiable risk factor incidence was assessed per module using generalized linear mixed models (GLMMs) with a random intercept per patient. Fixed effects included time (baseline, 3, 6, and 12 months), treatment group, and their interaction (time × group), with the primary inference based on the interaction to evaluate trajectory differences. A trajectory is defined as the longitudinal pattern of change in the outcome over time within each group. Models were fitted using maximum likelihood estimation with a missing-at-random assumption. Between-group differences at individual timepoints were assessed using model-based pairwise contrasts with Holm-adjusted *P*-values.

Given the dichotomous nature of the outcomes and the resulting limited within-subject variability, post hoc exploratory analyses were performed to evaluate the number of stable trajectories and between-group differences at individual timepoints using chi-square or Fisher’s exact tests. These analyses were unadjusted and considered complementary.

Health-related quality of life (EQ-5D-5L utility scores) was analysed using linear mixed-effects models adjusted for baseline differences. Due to the continuous nature of this outcome, no additional post hoc analyses were required.

All analyses were performed in IBM SPSS Statistics version 28.0 and RStudio, with a 2-sided *P*-value < .05 considered statistically significant.

## Results

### Baseline characteristics

**Figure 1.**
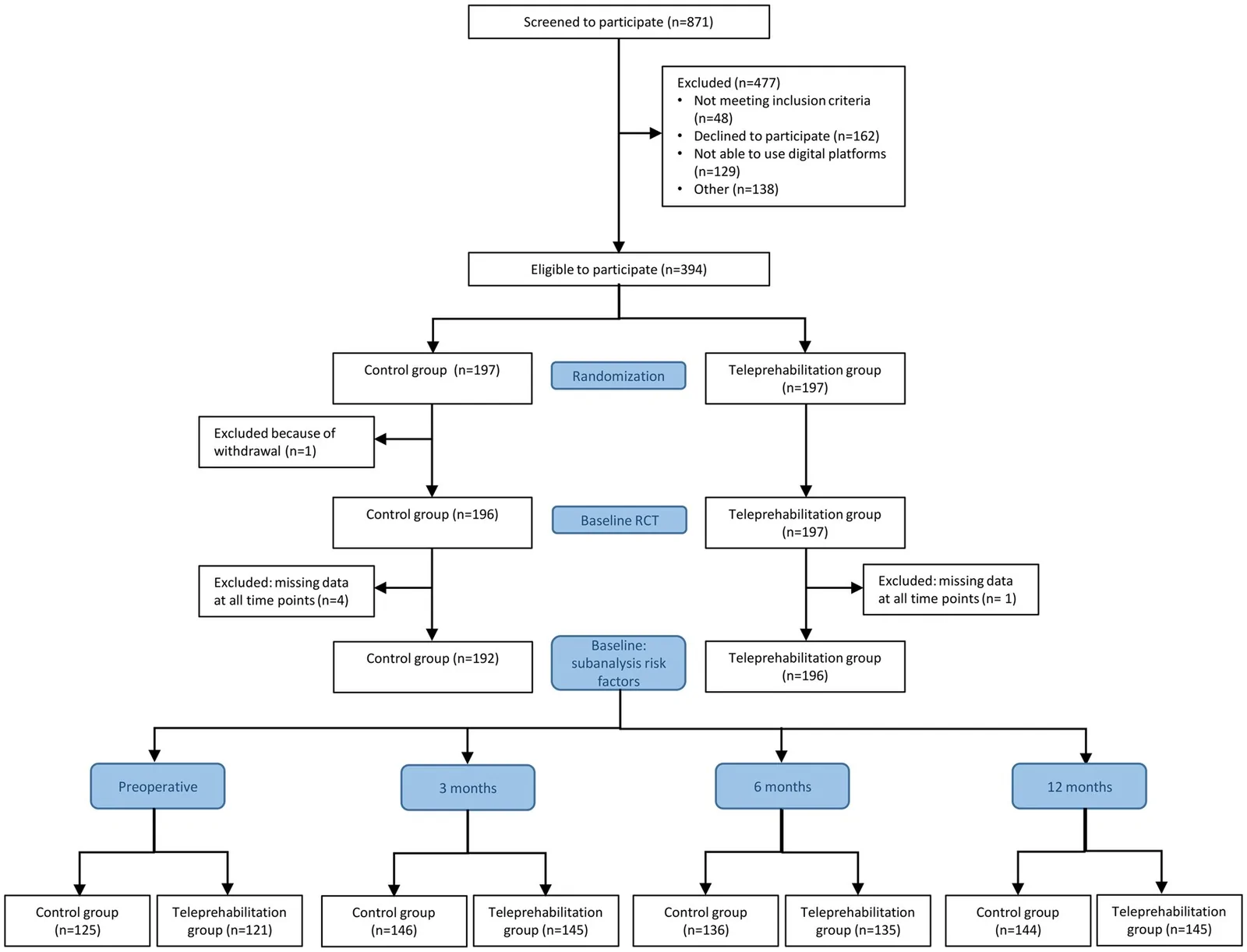
Flowchart Indicating the Number of Patients Assessed at Each Timepoint.

Data were prospectively registered between May 2020 and November 2023. Of 393 patients analysed in the DCC trial, 5 patients were excluded due to missing data at all follow-up timepoints, leaving 196 in the teleprehabilitation group and 192 in the control group. The study flowchart is presented in **Figure 1**.

Baseline characteristics are shown in **Table 1** and were comparable between groups. Patients had a mean age of 65.5 years, were predominantly male (75.8%), and had a median EuroSCORE II of 1.11 [0.74-1.72]. Coronary artery disease was the primary pathology (42.8%). The intervention group had a significantly lower CCS class and less frequently underwent minimally invasive surgery. No other significant differences were observed.

**Table 1.** Baseline Characteristics

	Overall (*n* = 388)	Intervention group (*n* = 196)	Control group (*n* = 192)	*P*-value
Demographic characteristics				
Age, years	65.5 ± 9.2	65.7 ± 9.3	65.4 ± 9.2	.523
Male	294 (75.8)	149 (76.0)	145 (75.5)	.909
Socioeconomic status	−0.029 ± 0.211	−0.014 ± 0.207	−0.046 ± 0.216	.416
Health-related characteristics				
BMI, kg/m^2^	27.1 ± 4.5	27.4 ± 4.4	27.7 ± 4.6	.580
EuroSCORE II	1.11 [0.74-1.72]	1.16 [0.77-1.73]	1.06 [0.72-1.70]	.289
Diabetes mellitus	74 (19.1)	36 (18.4)	38 (19.8)	.721
COPD	33 (8.5)	16 (8.2)	17 (8.9)	.807
Left ventricular ejection fraction				.510
<50	51 (13.1)	22 (11.2)	29 (15.1)	
≥50	337 (86.8)	174 (88.8)	163 (84.9)	
eGFR (mL/min/1.73 m^2^)	88.2 ± 29.3	88.5 ± 29.1	87.9 ± 29.4	.632
CCS class				.029
I	218 (56.2)	122 (62.2)	96 (50.0)	
II	136 (35.1)	64 (32.7)	72 (37.5)	
III or IV	34 (8.7)	10 (5.1)	24 (12.5)	
NYHA class				.472
I	86 (22.2)	48 (24.5)	38 (19.8)	
II	171 (44.1)	86 (43.9)	85 (44.3)	
III or IV	131 (33.8)	62 (31.6)	69 (36.0)	
Surgery characteristics				
Surgery type				.043
Conventional (sternotomy)	212 (54.6)	117 (59.7)	95 (49.5)	
Minimally invasive surgery (non-sternotomy)	176 (45.4)	79 (40.3)	97 (50.5)	
Primary pathology				.093
Coronary	166 (42.8)	83 (42.3)	83 (43.2)	
Valve	146 (37.6)	75 (38.3)	71 (37.0)	
Combined or other	76 (19.6)	38 (19.4)	38 (19.8)	
Centre				.615
MUMC+	104 (26.8)	50 (25.5)	54 (28.1)	
Zuyderland	195 (50.3)	101 (51.5)	94 (49.0)	
VieCuri	40 (10.3)	17 (8.7)	23 (12.0)	
Laurentius	40 (10.3)	22 (11.2)	18 (9.4)	
Other	9 (2.3)	6 (3.1)	3 (1.6)	

Data are presented as *n* (%), median [IQR], or mean ± SD, as appropriate.

Abbreviations: BMI, body mass index; CCS, Canadian Cardiovascular Society; COPD, chronic obstructive pulmonary disease; eGFR, estimated glomerular filtration rate; HADS, hospital anxiety and depression scale; IQR, interquartile range; NYHA, New York Heart Association.

### Effect of teleprehabilitation on the long-term risk factor incidence

#### Reduced physical fitness

**Figure 2.**
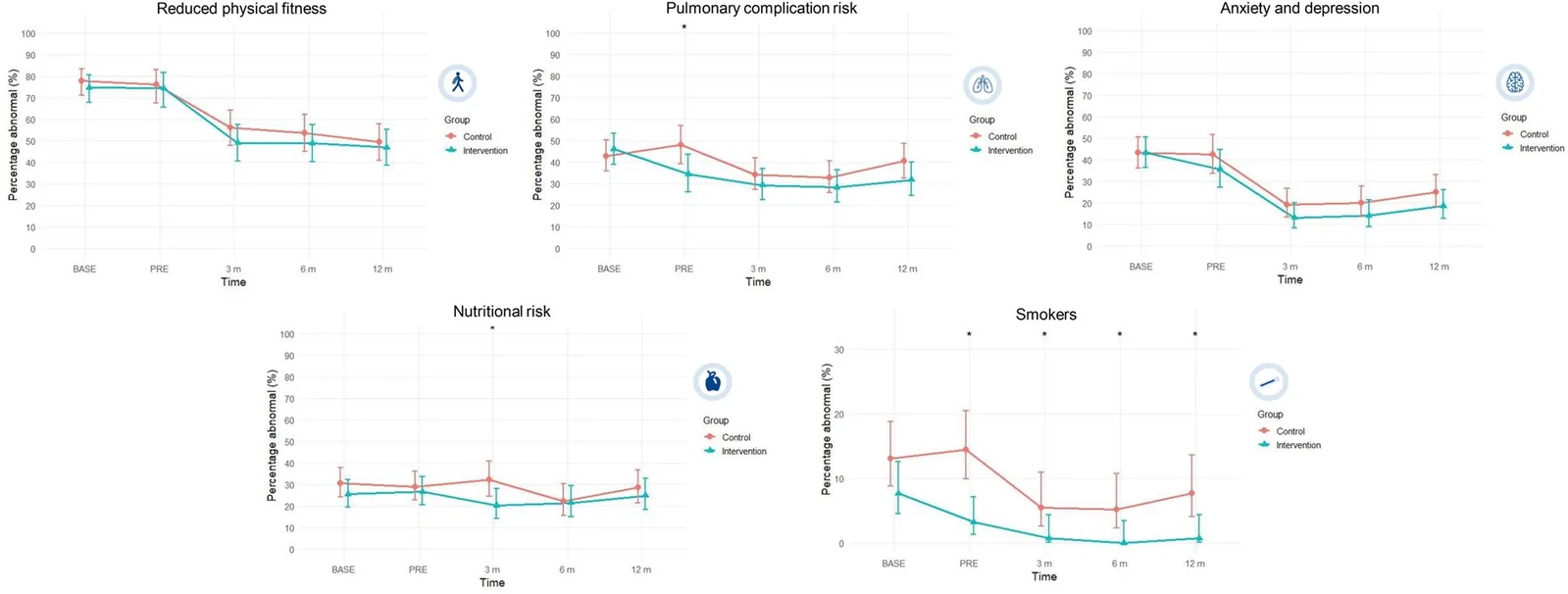
Observed Percentages of Each Risk Factor Per Module Over Time in Both Groups. *Indicates statistical difference (*P* < .05) based on unadjusted chi-square test per time.

We found a significant effect of time on reduced physical fitness incidence (*P* < .001), but no group × time interaction (*P* = .972) and no between-group differences at any timepoint. The proportion of patients with a stable course was similar in the intervention (50%) and control groups (53.1%); *P* = .607. Observed incidences decreased in both groups from approximately 75% preoperatively to 50% postoperatively, with no significant between-group differences (**Table 2**, **Figure 2**).

**Table 2.** The Observed Incidence of a Risk Factor Per Module at 3, 6, and 12 Months in Both Groups

	Observed incidence risk factor at 3 months			Observed incidence risk factor at 6 months			Observed incidence risk factor at 12 months		
	Intervention group (*n* = 145)	Control group (*n* = 146)	*P*-value	Intervention group (*n* = 135)	Control group (*n* = 136)	*P*-value	Intervention group (*n* = 145)	Control group (*n* = 144)	*P*-value
Risk factor									
Reduced physical fitness	71 (49.0)	82 (56.2)	.266	66 (48.9)	73 (53.7)	.505	68 (46.9)	71 (49.3)	.770
Pulmonary complications risk	48 (33.1)	55 (37.7)	.488	39 (28.9)	52 (38.2)	.133	47 (32.4)	59 (41.0)	.165
Anxiety and/or depression	19 (13.1)	28 (19.2)	.212	19 (14.1)	27 (19.9)	.269	27 (18.6)	36 (25.0)	.242
Nutritional risk	28 (19.3)	45 (30.8)	.033	29 (21.5)	30 (22.1)	1.000	36 (24.8)	41 (28.5)	.570
Smoking	1 (0.7)	8 (5.5)	.036	0 (0.0)	7 (5.1)	.014	1 (0.7)	11 (7.6)	.003

Data are presented as *n* (%).

#### Pulmonary complications risk

There was a significant change over time in the incidence of patients who had an elevated risk of postoperative pulmonary complications (*P* < .001), but no group × time interaction (*P* = .115) and no between-group differences at individual timepoints. A stable pattern was present in 71.4% of intervention and 75% of control patients (*P* = .729). Observed percentages decreased postoperatively in both groups, remaining at approximately 45% in the intervention group and 55% in the control group, without significant between-group differences (**Table 2**, **Figure 2**).

#### Anxiety and/or depression

A significant change in the incidence of increased anxiety and/or depression over time was observed (*P* < .0001), but no group × time interaction (*P* = .567) and no between-group differences at individual timepoints. A stable course was observed in 60.2% of intervention and 62.0% of control patients (*P* = .799). Incidences decreased in both groups after surgery, with slightly higher observed incidences in the control group, but no significant between-group differences in exploratory analyses (**Table 2**, **Figure 2**).

#### Nutritional risk

No significant change over time in the incidence of nutritional risk was observed in the GLMM (*P* = .173), but a significant group × time interaction (*P* = .011) indicated differing trajectories. Pairwise contrasts demonstrated a significant between-group difference at 3 months (odds ratio [OR] 11.2, 95% confidence interval [CI] 1.7-73.4; *P* = .012). Nutritional status stability was similar between groups (control: 82.8% versus intervention: 84.7%; *P* = .715). At 3 months, the proportion of patients with insufficient nutritional status was significantly lower in the intervention group (20.3% versus 32.1%; *P* = .036), driven by a higher malnutrition incidence in the control group (9% versus 2%; *P* = .038). No between-group difference in obesity incidence was observed at 3 months (*P* = .516), and nutritional risk did not differ significantly at later timepoints, remaining approximately at 30% in both groups (**Table 2**) (**Figure 2**).

#### Smoking

A significant change in smoking incidence over time (*P* < .001) and a significant time × group interaction (*P* = .045) were found, although pairwise contrasts showed no significant differences at individual timepoints. The behavioural pattern was significantly more stable in the intervention group (93.9%) than the control group (87.5%; *P* = .047). Smoking prevalence decreased in both groups after the cardiac surgery: in the control group, from 15% preoperatively to 5% postoperatively, with a slight increase to 7.6% at 12 months postoperatively; in the intervention group from 4% preoperatively to 0.7% postoperatively, reaching 0% at 6 months. Observed smoker percentages were significantly lower in the intervention group at 3, 6, and 12 months in exploratory chi-squared analysis (*P* < .05) (**Table 2**, **Figure 2**).

### Effect of teleprehabilitation on the quality of life over time

**Figure 3.**
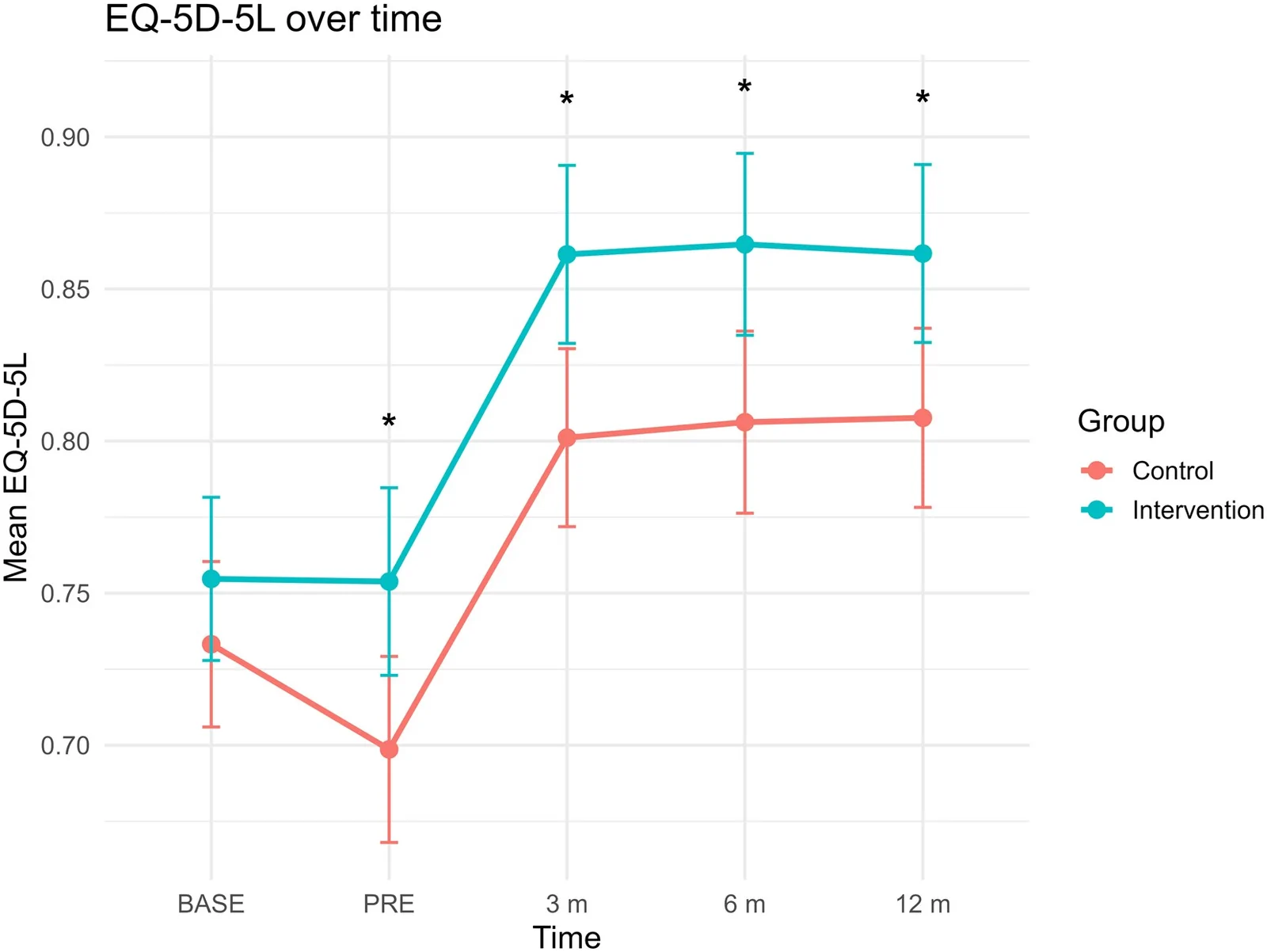
Estimated Marginal Means of EQ-5D-5L Utility Scores Over Time by Group, Based on a Generalized Linear Mixed Model. *P < .05.

After adjustment for baseline differences, a significant change in EQ-5D-5L scores (*P* < .001) was found over time and a significant group effect (*P* = .002), indicating overall quality of life improvement and higher scores in the intervention group. The time × group interaction was not significant (*P* = .219). EQ-5D-5L scores in the teleprehabilitation group remained stable preoperatively, whereas a decline was observed in the control group. After surgery, scores increased in both groups: from 0.703 to 0.806 in the control group and from 0.750 to 0.858 in the intervention group (**Figure 3**). Pairwise contrasts showed significantly higher adjusted EQ-5D-5L scores in the intervention group at all postoperative timepoints, with mean differences from 0.046 to 0.051 (*P* < .05) (**Supplemental material**).

## Discussion

In this secondary analysis of the DCC trial, we assessed the long-term effect of teleprehabilitation on modifiable risk factors and quality of life up to 1 year after elective cardiac surgery. Both groups showed significant postoperative reductions in all assessed risk factors except nutritional risk. Teleprehabilitation was associated with more favourable trajectories for smoking and nutritional risk throughout follow-up. Smoking prevalence remained lower at all timepoints, indicating sustained motivation to maintain smoking cessation over the 1-year follow-up period. The nutritional benefit at 3 months, driven by greater malnutrition in the control group, is consistent with the hypermetabolic and hypercatabolic stress response to cardiac surgery.^23^ Preoperative nutritional optimization may have mitigated this deterioration. Quality-of-life scores were consistently higher in the teleprehabilitation group and exceeded minimal clinically important difference thresholds.^24^ These findings suggest that while cardiac surgery acts as a motivational trigger for risk factor improvement, teleprehabilitation enhances these effects and contributed to sustained benefit.

### Modifiable risk factors

No significant between-group differences were observed in physical fitness, anxiety and/or depression, or pulmonary risk. The absence of a physical fitness effect contrasts with previous studies reporting significant improvements.^25^ However, in those studies, interventions were tailored to baseline physical performance and evaluated using objective measures. The lack of effect in the present study may therefore be attributable to the absence of objective baseline physical assessments, limiting the ability to individualize training intensity. Consequently, patients may have been insufficiently challenged. Moreover, the absence of preoperative differences between groups inherently limits the potential for divergent postoperative recovery trajectories.^12^ These findings suggest that the effectiveness of prehabilitation in improving physical fitness may depend on adequate baseline assessment and the implementation of individually tailored exercise prescriptions.

The absence of a between-group difference in pulmonary complications risk may reflect limitations of the composite score itself.^19^ Since only a limited number of its components are modifiable through prehabilitation, the score’s sensitivity to detect intervention effects may be restricted. In addition, more targeted outcome measures and objective physical assessments would possibly better capture pulmonary benefits.^19^^,^^26^

Lastly, the lack of significant effect on anxiety and depression contrasts with findings from studies in patients with elevated baseline anxiety or depression.^14^ In the present cohort, only a subset of patients had HADS scores above the threshold and received a targeted psychological intervention. Consequently, any treatment effect may have been diluted at the group level, obscuring between-group differences. It is also plausible that psychological benefits are most pronounced in the preoperative phase, as presented elsewhere,^12^ and attenuate after surgery.

Taken together, these findings suggest that further optimization of the teleprehabilitation programme may be achieved through more targeted patient screening, baseline objective assessment, and appropriate outcome assessment.

### Quality of life

Patients receiving teleprehabilitation demonstrated clinically meaningful higher quality of life from preoperative screening up to 1 year after surgery. This sustained benefit contrasts with previous cardiac prehabilitation studies reporting no effect on short-term postoperative quality of life^9^^,^^27^^,^^28^ and may be explained by the multimodal nature of the intervention. By addressing psychological health alongside physical training, nutritional status, and smoking, teleprehabilitation targets multiple determinants of recovery. Evidence suggests that addressing psychological health can specifically enhance postoperative quality of life,^29^ supporting the hypothesis that a multimodal approach contributes to more sustained improvements. The absence of a significant time × group interaction reflects that between-group differences were established during the preoperative phase and preserved, rather than widened, throughout postoperative follow-up.

### Strengths and limitations

A strength of this study is its multicentre randomized design with extended follow-up. Several limitations should be noted. First, missing data were present at all timepoints and may have introduced selection bias. Although GLMMs handle missing data at random, violation of this assumption cannot be excluded. Second, due to a technical error, VAS data for physical fitness were unavailable for the intervention group at all postoperative timepoints. However, given the absence of preoperative between-group differences and the comparable pattern across physical fitness outcomes, we do not expect this to have substantively influenced the results. Third, as a secondary analysis, the study was not powered to detect differences in individual risk factors, increasing the risk of type II error, particularly for low-incidence outcomes such as smoking. In addition, the limited within-subject variability observed during follow-up may have resulted in conservative estimates within the generalized linear mixed model. Fourth, all outcome measures were patient-reported, introducing the potential for recall bias. Fifth, higher CCS class and more minimally invasive surgery in the control group could theoretically influence motivation and surgical impact, but are unlikely to have affected the results. Sixth, both groups received access to an information platform and completed questionnaires, which may have motivated control patients to address their risk factors, potentially attenuating between-group differences through a Hawthorne effect. Finally, participation required digital literacy, which may limit generalizability; however, structured digital onboarding and technical support could mitigate this barrier in broader implementation.

### Future perspectives

Future studies should prospectively evaluate the effects of teleprehabilitation on individual modifiable risk factors with sufficient power to detect subgroup-specific effects, particularly in patients with elevated baseline risk. Incorporating objective outcome measures, such as physical performance tests and objective measures of physical fitness and inspiratory muscle strength, would strengthen future evaluations. A hybrid prehabilitation approach, combining remote delivery with supervised in-person components, particularly for physical training and inspiratory muscle training, may enhance intervention effectiveness and warrants prospective investigation. Strategies to extend structured support into the postoperative period should also be explored to promote sustained behavioural change.

### Conclusion

Teleprehabilitation was associated with more favourable postoperative trajectories in smoking behaviour and nutritional risk, and clinically meaningful improvements in quality of life up to 1 year after elective cardiac surgery. While cardiac surgery acts as a motivational trigger for improvements in modifiable risk factors, the addition of a structured, multimodal teleprehabilitation programme may enhance these effects and contribute to more sustained behavioural change. These findings support teleprehabilitation as a potentially valuable addition to standard preoperative care, with future research incorporating objective physical assessments needed to further clarify long-term effects on physical outcomes.

## Supplementary Material

ezag207_Supplementary_Data

## Data Availability

The data underlying this article will be shared on reasonable request to the corresponding author.
