# Supplementary material for: The Long-Term Impact of a Teleprehabilitation Programme on Modifiable Risk Factors and Quality of Life After Cardiac Surgery
Source: Eur J Cardiothorac Surg. 2026 Aug 6;68(8):ezag207. doi: 10.1093/ejcts/ezag207 (PMC13501530; doi:10.1093/ejcts/ezag207)
Supplement: Supplementary file 1 [file ezag207_supplementary_data.zip › Supplemental material.docx]

**SUPPLEMENTAL MATERIAL**

*Table 1*. Estimated marginal means of EQ-5D-5L, adjusted for baseline differences, and mean differences between intervention and control groups over time.

|  | **Control group (n=192)** | **Intervention group (n=196)** | **Mean difference (intervention - control)** | **P-value** |
| --- | --- | --- | --- | --- |
| **Time point** | | | | |
| Prior to surgery | 0.703  (0.678- 0.729) | 0.750  (0.725 - 0.776) | 0.047  (0.011- 0.083) | 0.011 |
| 3 months after surgery | 0.806  (0.783- 0.829) | 0.858  (0.835- 0.881) | 0.050  (0.017– 0.084) | 0.003 |
| 6 months after surgery | 0.809  (0.786- 0.833) | 0.864  (0.840- 0.887) | 0.051  (0.016– 0.085) | 0.004 |
| 12 months after surgery | 0.812  (0.789- 0.835) | 0.861  (0.839- 0.884) | 0.046  (0.012– 0.079) | 0.008 |

Data are presented as mean (95% CI).
